# A DFT study of bandgap tuning in chloro-fluoro silicene

**DOI:** 10.1039/d3ra07452h

**Published:** 2024-02-06

**Authors:** Uzair Khan, M. Usman Saeed, Hosam O. Elansary, Ihab Mohamed Moussa, Aziz-Ur-Rahim Bacha, Y. Saeed

**Affiliations:** a Department of Physics, Abbottabad University of Science and Technology Abbottabad KPK Pakistan yasir.saeed@kaust.edu.sa yasirsaeedphy@aust.edu.pk +(92)-3454041865; b Department of Plant Production, College of Food Agriculture Sciences, King Saud University Riyadh 11451 Saudi Arabia; c Department of Botany and Microbiology, College of Science, King Saud University P.O. Box 2455 Riyadh 11451 Saudi Arabia; d State Key Laboratory of Urban Water Resource and Environment, Shenzhen Key Laboratory of Organic Pollution Prevention and Control, School of Civil and Environmental Engineering, Harbin Institute of Technology Shenzhen Shenzhen 518055 P. R. China

## Abstract

The structural, electronic and optical properties of silicene and its derivatives are investigated in the present work by employing density functional theory (DFT). The Perdew–Burke–Ernzerhof generalized gradient approximation (PBE-GGA) is used as the exchange–correlation potential. Our results provide helpful insight for tailoring the band gap of silicene *via* functionalization of chlorine and fluorine. First, relaxation of all the materials is performed to obtain the appropriate structural parameters. Cl–Si showed the highest lattice parameter 4.31 Å value, while it also possesses the highest buckling of 0.73 Å among all the derivatives of silicene. We also study the electronic charge density, charge difference density and electrostatic potential, to check the bonding characteristics and charge transfer between Si–halides. The electronic properties, band structures and density of states (DOS) of all the materials are calculated using the PBE-GGA as well as the modified Becke–Johnson (mBJ) on PBE-GGA. Pristine silicene is found to have a negligibly small band gap but with the adsorption of chlorine and fluorine atoms, its band gap can be opened. The band gap of Cl–Si and F–Si is calculated to be 1.7 eV and 0.6 eV, respectively, while Cl–F–Si has a band gap of 1.1 eV. Moreover, the optical properties of silicene and its derivatives are explored, which includes dielectric constants *ε*_1_ and *ε*_2_, refractive indices *n*, extinction coefficients *k*, optical conductivity *σ* and absorption coefficients *I*. The calculated binding energies and phonon band structures confirm the stability of Cl–Si, Cl–F–Si, and F–Si. We also calculated the photocatalytic properties which show silicine has a good response to reduction, and the other materials to oxidation. A comparison of our current work to recent work in which graphene was functionalized with halides, is also presented and we observe that silicene is a much better alternative for graphene in terms of semiconductors and photovoltaics applications.

## Abstract 2

The structural, electronic and optical properties of silicene and its derivatives are investigated in the present work by employing density functional theory.

## Graphical abstract

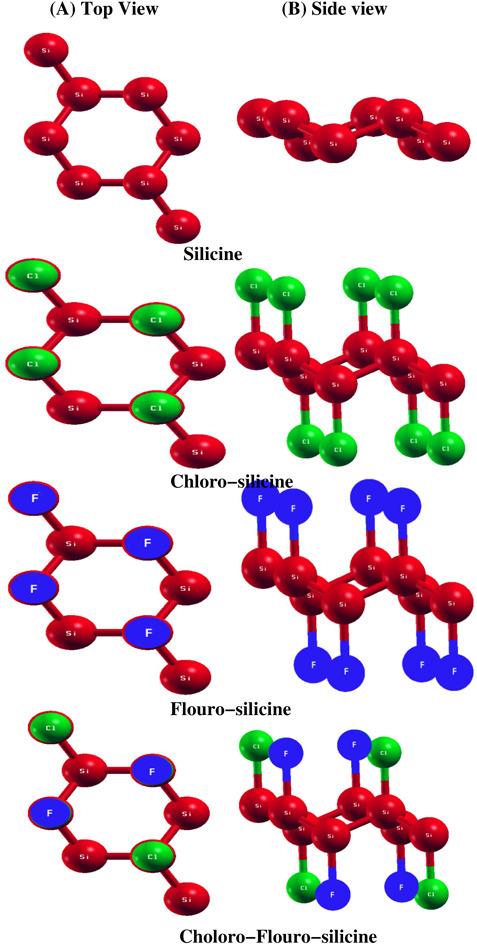

## 1. Introduction

After the discovery of graphene (2D layer of carbon atoms) in 2004,^1^ other Group IV elements received attention, particularly silicene (2D buckled layer of silicon atoms). The possibility that silicene could be a replacement for graphene is one of the main reasons why it is of interest. Contrary to graphene, silicene possesses sp^2^ and sp^3^ hybridization of silicon atoms, which is why it has a buckled structure.^2^ Properties like high charge carrier mobility, greater spin–orbit coupling and tunable band gap make it superior to graphene.^3–5^ Although silicene is one of the most promising candidates for electronic devices, it is not widely used due to its negligible band gap around the Fermi level.^6^ Opening the band gap in silicene would lead to wonderful applications in electronic devices, especially in photovoltaics, supercapacitors and FETs.^7^ However, doped-stanene monolayers have been studied theoretically; Al-doped, B-doped, N-doped and P-doped stanene monolayers show metallic characteristics for optoelectronic applications. Also stanene monolayers have been studied for gas sensing applications for gases including CO, NO, N_2_O and NH_3_.^8–16^

For tuning the band gap of silicene, various approaches have been used. The oxidation of silicene was done to determine the likelihood of silicene oxide formation and how oxidation would affect the physical characteristics of silicene.^17,18^ These works showed that on free-standing silicene, the O_2_ molecule splits into O atoms, resulting in the production of the complex Si–O. Numerous researches have looked at the chemical reactivity of 2D materials toward different adsorbing gas molecules. In 2021, the dissociative adsorption of NH_3_, PH_3_, H_2_O, and H_2_S on graphene and silicene was studied using density functional theory.^19^ On the silicene surface, nearly all dissociative adsorptions were exothermic, whereas those on the graphene surfaces were endothermic. This work showed that silicene is more responsive to dissociative adsorption than graphene. Applying a perpendicular electric field to silicene’s two sublattices was proposed as a feasible way to make a band gap in silicene without damaging its electrical characteristics.^20^ Nguyen *et al.*^21^ show that Cl–Si = 2 : 2 and F–Si = 2 : 2 had non-magnetic behaviour while odd ratio Si–halides show small magnetic moments.

The vast physical and chemical properties of transition-metals adsorbed or doped onto silicene have long attracted the curiosity of researchers. Silicene has remarkable electrocatalytic activity and a variety of electrical properties when adsorbed or doped with transition metals.^22^ Recently, a DFT investigation of silicene doped with metal (Al, Ag, and Au) substrates of various thicknesses was performed.^23^ Due to the high cost of the substrate materials, the silicene produced on silver and gold substrates is not suitable for wide-scale use. Another advantage of silicene’s chemically active surface is that it may expand its band gap through chemical functionalization processes like hydrogenation; the hydrogen stabilises silicene by coating its surface. It is anticipated that when silicene is completely hydrogenated to form silicane, wide band-gap (from 0 to 3 eV) semiconductors would be created.^24^ Additionally, utilising the quantum confinement effect, DFT studies predict adjustable band gaps in silicene nanoribbons up to about 0.4 eV.

The effect of functionalization of Cl and F on graphene have been studied recently, leading to the formation of derivatives of graphene *i.e.*, chloro–graphene (Cl–C), fluoro–graphene (F–C) and chlorofluoro–graphene (Cl–F–C).^25^ This work showed that the derivatives of graphene have a wide variety of structural, electrical, and optical properties. However the band gap values of all the derivatives of graphene are quite high (ranging from 2.9 eV to 4.9 eV), which make them unsuitable for semiconducting applications.

In our present work, we have replaced graphene with silicene, which is considered as a potential candidate for semiconductor applications.^26^ We performed the functionalization of Cl and F on silicene, resulting in the formation of derivatives of silicene, *i.e.* chloro–silicene (Cl–Si), fluoro–silicene (F–Si) and chlorofluoro–silicene (Cl–F–Si). Then we investigated the structural, electronic and optical properties of silicene and its derivatives by employing density functional theory (DFT).

## 2. Computational details

We used the WIEN2k code in our calculations^27^ using density functional theory (DFT),^28^ to calculate quantum mechanical issues linked to solid-state electronic systems, supported by the Linux operating system. We used the full potential linearized augmented plane wave plus local orbital (FP-LAPW + Lo) method for calculations.^29^ We used the more precise exchange–correlation potential PBE-GGA as a generalized gradient approximation to analyze the band structure of silicene and its derivatives.^30^ However, for chloro-fluorinated-silicene, a 2 × 2 × 1 super cell (16 atoms) was employed to study structural arrangements. In order to obtain the correct band gap, the modified Becke–Johnson (mBJ) over the PBE-GGA was used. For relaxation and scf calculations, a 16 × 16 × 1 *k*-mesh is used for Si, Cl–Si, F–Si and Cl–F–Si. All calculations are performed using an energy tolerance of 10^−5^ Ryd. A 20 Å vacuum slab is used to prevent artificial interaction with periodic images due to the employed periodic boundary conditions. The charge density difference, electrostatic potential and phonon dispersion calculations are performed using the generalized gradient approximation of the exchange–correlation potential in the Perdew–Burke–Ernzerhof as implemented in the Quantum ESPRESSO code.^31^ We employ a plane wave energy cutoff of 550 eV and a *k*-mesh of 8 × 8 × 1 for the Brillouin zone integrations.

## 3. Results and discussion

### 3.1 Structural properties

**Fig. 1:**
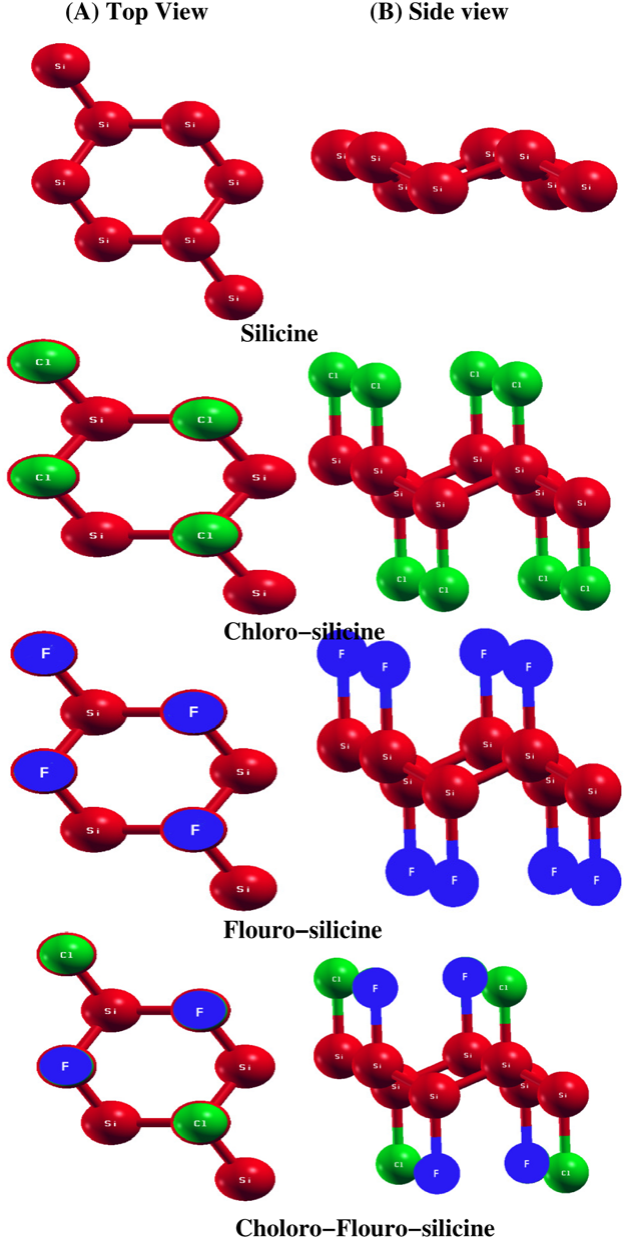
Structure of Si, Cl–Si, F–Si and Cl–F–Si.

The structural properties of a material decide its actual application. Here, we study the structural properties of Si and its derivatives Cl–Si, F–Si and Cl–F–Si. We calculated three important structural parameters, *i.e.* lattice parameters, distance between Si atoms and buckling of Si atoms, by first performing relaxation of all the above mentioned structures. Fig. 1 shows the monolayer of silicene. Unlike graphene, it has a buckled geometry. We first relaxed the silicene structure and calculated its lattice parameter as 3.86 Å and the bond length Si–Si is equal to 2.28 Å, which is in good agreement with previous experimental work^32^ as well as the theoretical studies.^33^ All the experimental and theoretical studies suggest that height of buckling in Si is about 0.45 Å, which is close to our calculated value, *i.e.* 0.48 Å. Moreover to avoid interactions between two adjacent monolayers, a vacuum space along the *z*-axis of 20 Å is chosen. In Table 1 we make a comparison between our calculated parameters and the previous experimental as well as theoretical values.

**Table 1:** Comparison of structural parameters from our present work with previous experimental and theoretical studies for Si: lattice constant (*a*), distance between Si atoms (*d*_Si–Si_) and buckling height

Parameters	*a* (Å)	*d*_Si–Si_ (Å)	Buckling (Å)
Experimental^32^	3.87	2.27	0.45
Theoretical work^33^	3.86	2.29	0.45
Present work	3.86	2.28	0.48

In Table 2, we present a comparison of the structural parameters of pristine silicene with its derivatives *i.e.* Cl–Si, F–Si and Cl–F–Si. A 2 × 2 × 1 supercell is used to study all structural parameters of the derivatives of silicene. Increase in lattice parameter and Si–Si distance was observed as we adsorbed fluorine and chlorine in silicene. Moreover, Cl–Si, F–Si and Cl–F–Si have buckling values of 0.73 Å, 0.71 Å and 0.70 Å, respectively. If we compare all the values, we find that the buckling of silicene increased with absorbed chlorine and fluorine, the highest buckling is observed in the Cl–Si.

**Table 2:** Comparison of the structural parameters of Si with its derivatives, namely lattice parameters (*a*), Si–Si distance (*d*_Si–Si_), Si–F distance (*d*_Si–F_), Si–Cl distance (*d*_Si–Cl_), Cl–Cl distance (*d*_Cl–Cl_) and F–F distance (*d*_F–F_), the value of the actual lattice parameter of the 2 × 2 × 1 unit cell is reduced by half to compare all values

Properties	Si	Cl–Si	Cl–F–Si	F–Si
*a* (Å)	3.86	4.31	4.12	4.00
*d*_Si–Si_ (Å)	2.28	2.36	1.75	2.35
*d*_Si–F_ (Å)	—	—	1.63	1.63
*d*_Si–Cl_ (Å)	—	2.08	2.08	—
*d*_Cl–Cl_ (Å)	—	5.39	2.91	—
*d*_F–F_ (Å)	—	—	2.91	4.57
Buckling (Å)	0.48	0.73	0.72	0.71

These findings demonstrate the wide diversity of structural characteristics of silicene derivatives. However, there is a significant fall in the lowest Si–Si distance, indicating non-linear behaviour rather than a smooth transition. There are four Cl-atoms and four F-atoms in a unit cell, which are above and below the Si-atoms. Each Cl-atom and F-atom is bonded to its nearest-neighbor Si atoms. This divergent pattern makes sense given that Cl’s covalent radius (0.99 Å) is much larger than that of F (0.71 Å).

### 3.2 Bonding characteristics

**Fig. 2:**
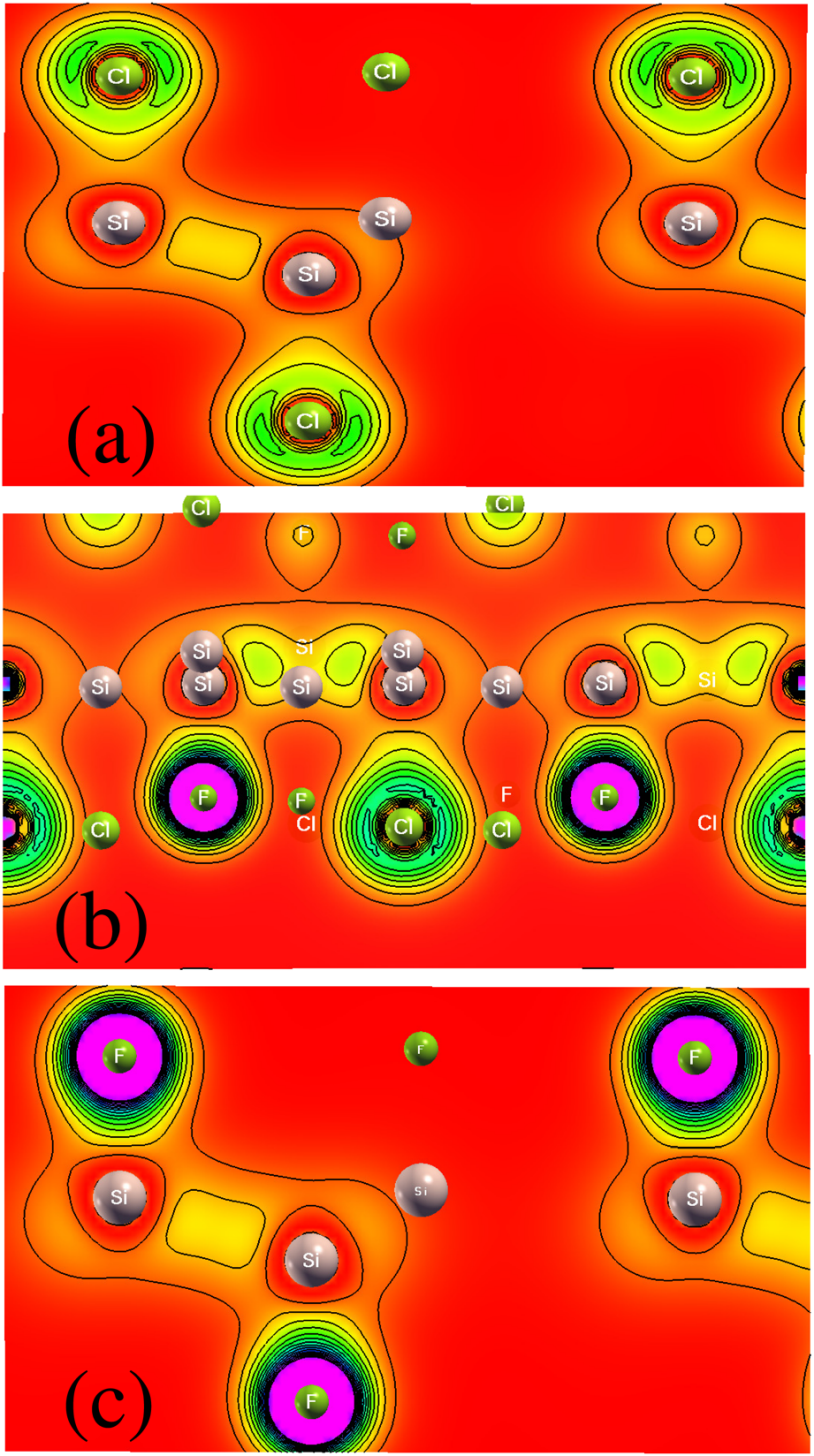
Charge density of (a) Cl–Si, (b) Cl–F–Si and (c) F–Si.

We investigated the two-dimensional (2D) electronic charge density contours for Cl–Si, Cl–F–Si and F–Si to analyze the origin of the Si–halide chemical bond, as shown in Fig. 2. It is found that the distribution of electronic charge is spherical, which results in the bonding between Si–halides showing the prevailingly ionic features due to the large electronegativity difference between Si (1.9), Cl (3.16) and F (3.98). This is probably because of the Cl and F p-state contribution to the valence band.

**Fig. 3:**
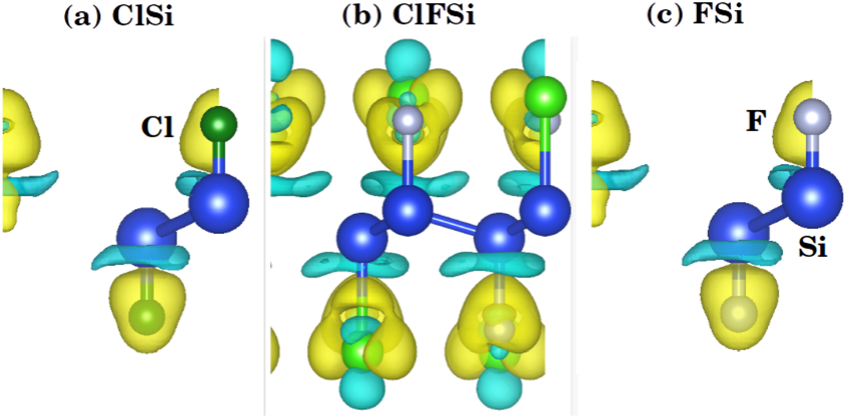
Isosurface plots of charge density difference of (a) Cl–Si, (b) Cl–F–Si and (c) F–Si.

To further investigate the charge transfer properties of Cl–Si, Cl–F–Si and F–Si, we calculated their charge density differences (CDD), as shown in Fig. 3. The yellow and cyan areas in Fig. 3 represent electron accumulation and depletion (isovalue is ±0.00573 a. u), respectively. It can be seen from Fig. 3(a–c) that charges were accumulated (electrons are transferred) to the Cl and F sites from the silicene monolayer.

### 3.3 Binding energy, electrostatics potential, and phonon dispersion

Binding energy is a standard to check the stability of a material. Binding energy is the amount of energy needed to disperse all of the particles in a system or to detach a particle from it. The binding energy of Cl–Si, Cl–F–Si and F–Si is calculated from eqn (1)–(3), respectively.1
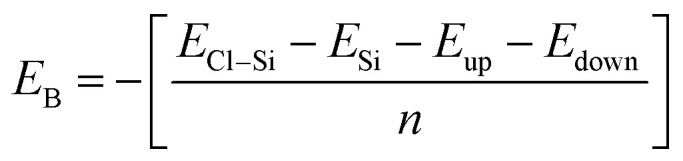
where, *E*_Cl–Si_ = total energy of Cl–Si, *E*_Si_ = energy of pristine silicene, *E*_up_ = sum of the energies of all upper Cl-atoms (without adsorption on Si), *E*_down_ = sum of the energies of all lower Cl-atoms (without adsorption on Si), *n* = total number of atoms being adsorbed (in our case, *n* = 8);2
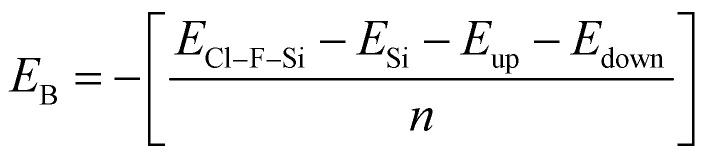
where, *E*_Cl–F–Si_ = total energy of Cl–F–Si, *E*_Si_ = energy of pristine silicene, *E*_up_ = sum of the energies of all upper F-atoms (without adsorption on Si), *E*_down_ = sum of the energies of all lower F-atoms (without adsorption on Si);3
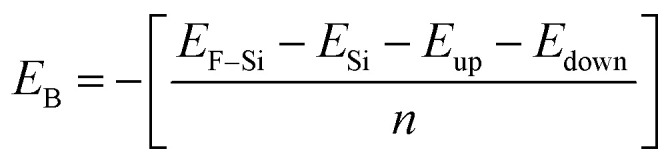
where, *E*_F–Si_ = total energy of F–Si, *E*_Si_ = energy of pristine silicene, *E*_up_ = sum of the energies of all upper F-atoms (without adsorption on Si), *E*_down_ = sum of the energies of all lower F-atoms (without adsorption on Si).

Higher stability results from a reduced binding energy. From Table 3, although all our materials are predicted to be stable, we can see that F–Si has the lowest binding energy which indicates that it is the most stable derivative of silicene of the three. The magnitude of binding energy is found to be increased with increasing chlorine concentration. Furthermore, due to its highly reactive buckling surface, in comparison to halogen-adsorbed graphene, halogen-absorbed silicene is more geometrically stable.^25^

**Table 3:** Calculated binding energies (*E*_B_) for all the derivatives of silicene

Materials	*E*_B_ (eV)
Cl–Si	−3.22631
Cl–F–Si	−4.71631
F–Si	−5.29490

**Fig. 4:**
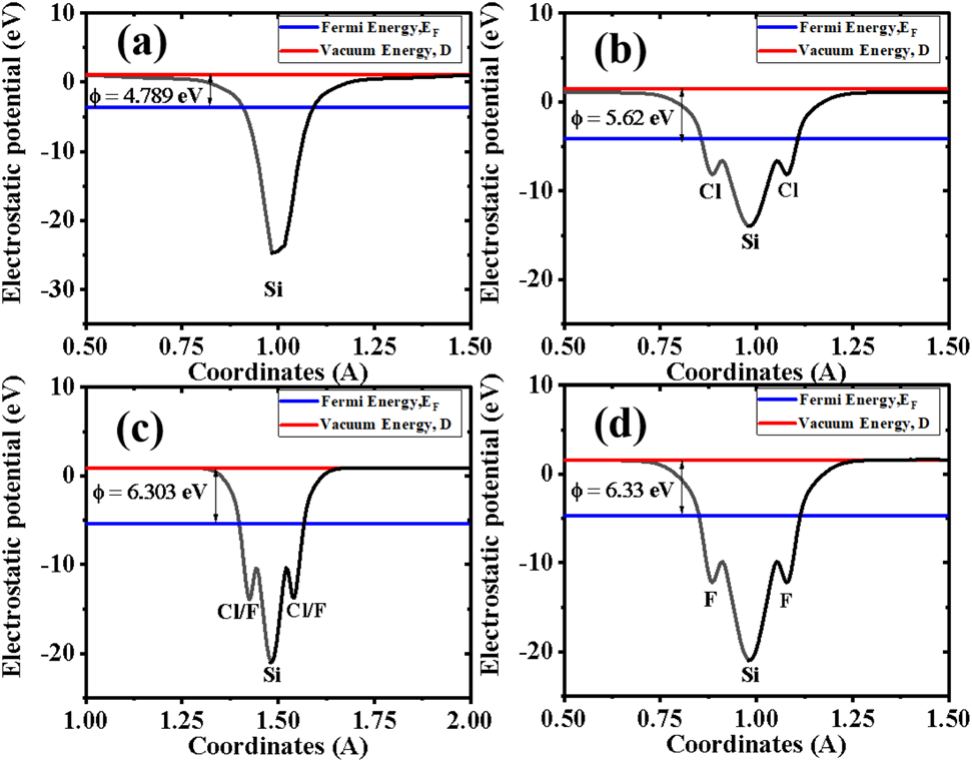
Electrostatic potential and work function of (a) Si, (b) Cl–Si, (c) Cl–F–Si and (d) F–Si.

The calculated electrostatic (electric) potential and work function of Si, Cl–Si, Cl–F–Si and F–Si are shown in Fig. 4(a–d), respectively. In all cases the silicene monolayer has a deeper potential of ∼25 eV than Cl/F which confirms that charges/electrons are transferred from the silicene monolayer to the Cl/F atoms, agreeing with the results obtained from charge density and CDD plots (Fig. 2 and 3, respectively). Additionally, we also calculated the work function (*ϕ*), which may be defined as the difference between the vacuum level and Fermi energy. The work function corresponds to the minimum amount of energy needed to remove an electron from the surface. The work function of Si, Cl–Si, Cl–F–Si and F–Si is 4.789, 5.62, 6.303 and 6.33 in eV, respectively. The large values of *ϕ* for Cl–Si, Cl–F–Si and F–Si compared to the silicene monolayer, are due to the introduction of Cl/F atoms.

**Fig. 5:**
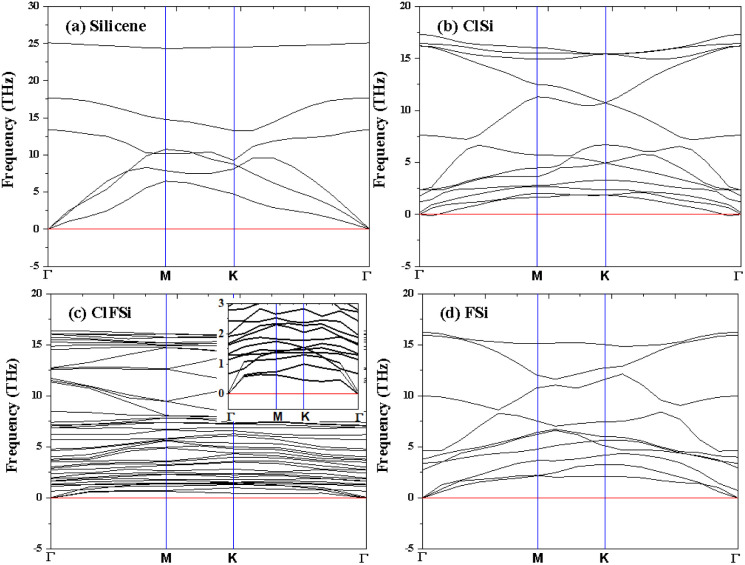
Phonon dispersion of (a) Si, (b) Cl–Si, (c) Cl–F–Si and (d) F–Si.

Binding energies are not sufficient to confirm the stability of Cl–Si, Cl–F–Si and F–Si, so we also calculated the phonon band structure of Cl–Si, Cl–F–Si and F–Si to further confirm the stability, see Fig. 5. All structures are dynamically stable with zero negative frequency bands.

### 3.4 Electronic band structure

**Fig. 6:**
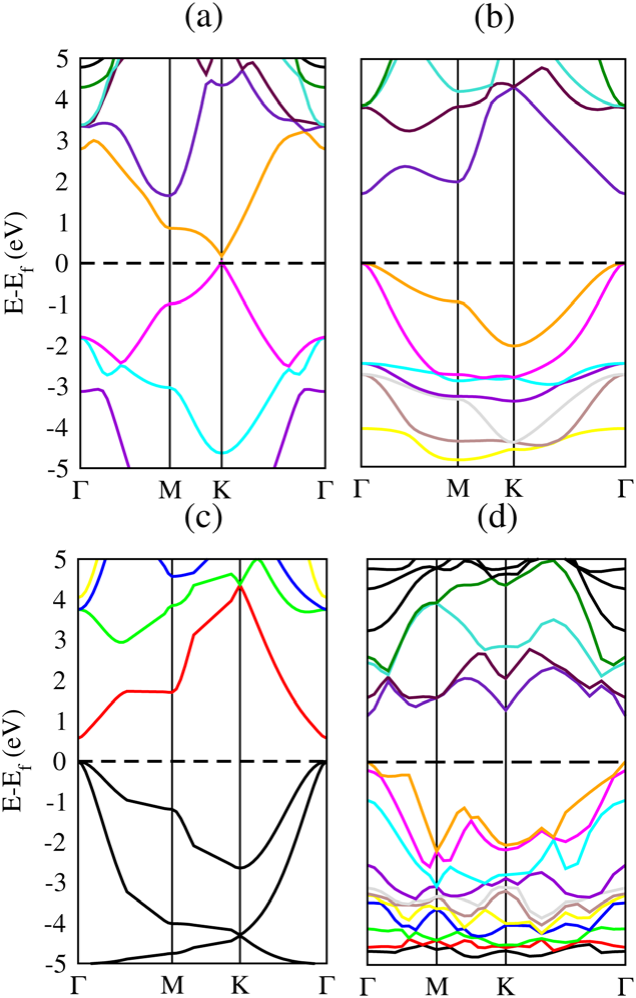
Band structures of (a) Si, (b) Cl–Si, (c) F–Si and (d) Cl–F–Si.

Band structure is very significant when studying the electronic behavior of materials. Metallic or semiconducting band structures can reveal the composition of materials. Physical properties of solids can easily be described by electronic band structure. These properties include optical behavior and electrical resistivity and it serves as a basis for devices like transistors, solar cells *etc.* Therefore, we have calculated the band structures to realize the electronic behavior of Si, Cl–Si, F–Si and Cl–F–Si as shown in Fig. 6(a)–(d), respectively. We used two different methods to quantify the band gap for all of the compounds. At first, we measured the band gap using a simple scf calculation. Secondly to get a more accurate value of the band gap, we performed calculations using the modified Becke–Johnson (mBJ) exchange potential method. The calculated band gap of all considered materials are given in Table 4. Those materials which possess an indirect band gap cannot be used in optoelectronic junction devices because of the phonon interaction, they are poor light emitters. All our materials have a direct band gap, so they can be utilized in optoelectronic devices.^35^

**Table 4:** Band gap values of silicene and its derivatives

Materials	*E*_g_ (eV) (PBE-GGA)	*E*_g_ (eV) (TB-mBJ)
Si	0	0.031, 0.027 (ref. 34)
Cl–Si	1.3	1.7
Cl–F–Si	0.4	1.1
F–Si	0	0.6

Next we make a comparison of silicene and its derivatives with graphene (C) and its derivatives: chloro–graphene (Cl–C), fluoro–graphene (F–C) and chlorofluoro–graphene (Cl–F–C).^25^ The band gap values of all the derivatives of graphene are higher (ranging from 2.9 eV to 4.9 eV), which makes it unsuitable for semiconducting applications. While our present work on derivatives of silicene provides band gaps ranging from 0.6 eV to 1.7 eV, which is suitable for many semiconductor devices and photovoltaic applications (Table 5).

Comparison of silicene and its derivatives with graphene and its derivatives

**Table:** 

Materials	*E*_g_ (eV) (TB-MBJ)
Si	0.031
Cl–Si	1.7
Cl–F–Si	1.1
F–Si	0.6

**Table:** 


Materials	*E*_g_ (eV) (HSE06)^25^
C	0
Cl–C	2.9
Cl–F–C	3.0
F–C	4.9

### 3.5 Density of states (DOS)

**Fig. 7:**
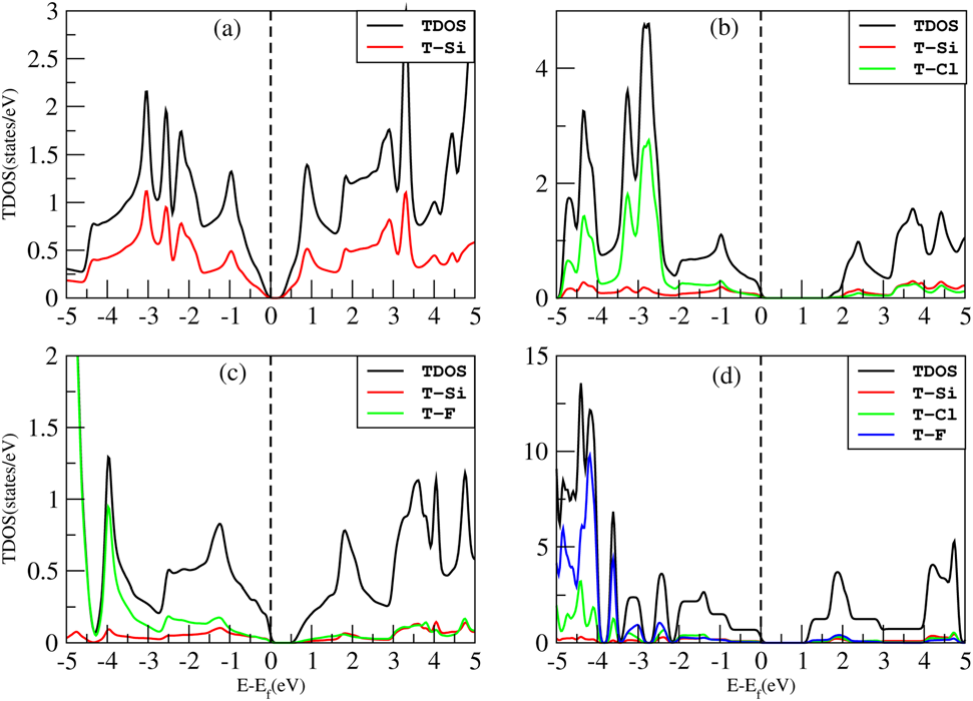
TDOS of (a) Si, (b) Cl–Si, (c) F–Si and (d) Cl–F–Si.

The density of states (DOS), or the number of electron states per unit volume per unit energy, is the number of different states that electrons are allowed to occupy at a particular energy level. We studied the DOS of our materials (Si, Cl–Si, F–Si and Cl–F–Si) in order to study the behavior of the band structure and the orbitals contributions to the valence band maxima (VBM) and conduction band minima (CBM).First we discuss the total density of states (TDOS) of all the materials using TB-MBJ on PBE-GGA as shown in Fig. 7. In the case of Cl–Si, Cl atoms provide a major contribution to the VBM while Si-atoms provide a major contribution to the CBM. As far as F–Si is concerned, F-atoms play a major role in both the VBM and CBM. Similarly, F-atoms have major contributions in the DOS of Cl–F–Si.

**Fig. 8:**
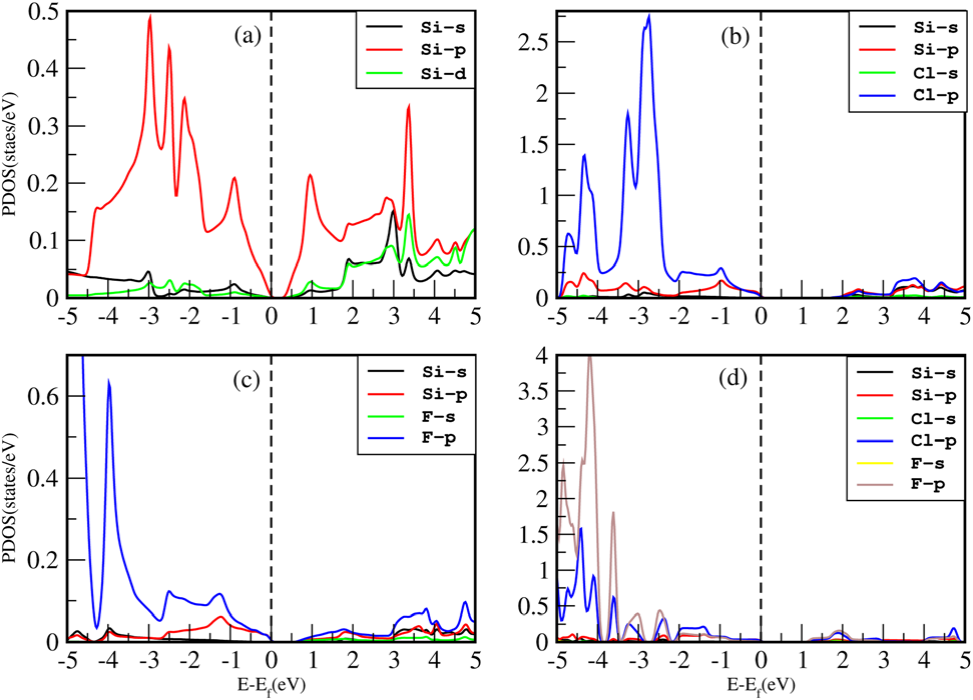
PDOS of (a) Si, (b) Cl–Si, (c) F–Si and (d) Cl–F–Si.

The impact of Cl and F concentration on the electronic structure is further elucidated in Fig. 8 which depicts the partial density of states (PDOS) of all the materials with TB-MBJ on PBE-GGA. In silicene, the p-orbital has a greater contribution to the VBM and CBM than all other orbitals. In the case of Cl–Si, the p-orbital of Cl and silicene atoms provides a major contribution to the VBM and CBM, and also shows hybridization near the Fermi level. A similar trend is observed for F–Si and Cl–F–Si, where the p-orbital of Cl/F/Si mostly contributes to the PDOS. All the hybridization near the Fermi level is between the p-states, and no s–p hybridization occurs which also confirms the ionic nature of these structures.

### 3.6 Optical properties

Light has a variety of interactions with matter. Optical characteristics are described as a substance’s reaction to electromagnetic radiation, and optical materials are substances whose properties may be influenced by light flow. Every substance has its unique optical qualities. Semiconductors are often opaque to visible light but transparent to ultraviolet light. The optical qualities of a substance are governed by its structural properties and chemical composition, which vary from one material to the next. Solid-state optical characteristics may be used to compute energy band structure, impurities, defects, and lattice vibrations. The nature of absorption, reflection, and transmission will be briefly reviewed in this section. The frequency dependent dielectric function *i.e. ε*(*ω*) = *ε*_1_ + i*ε*_2_(*ω*) is used to determine the optical properties. For cubic crystals the frequency-dependent real and imaginary part of the dielectric function *ε*_1_(*ω*) and *ε*_2_(*ω*), respectively, are given as^36^4
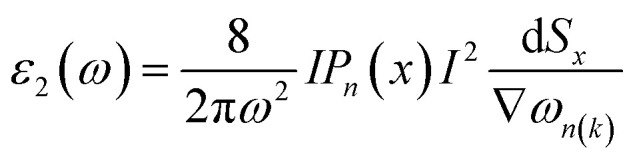
5
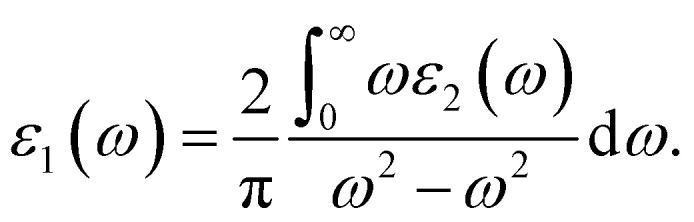


Optical properties like refractive indices, optical conductivity, reflectivity and absorption coefficients *etc.* can be calculated by the dispersion of real and imaginary components of dielectric features. We have discussed the optical properties of Si, Cl–Si, F–Si and Cl–F–Si in this section.

**Fig. 9:**
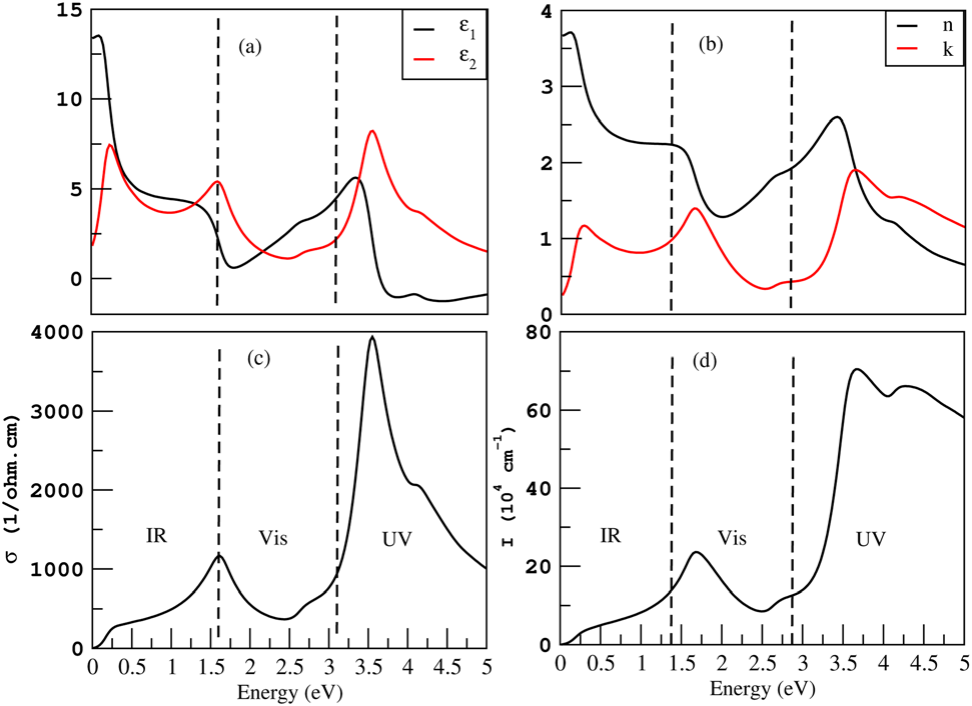
Optical properties of Si, (a) *ε*_1_ and *ε*_2_, (b) *n* and *k*, (c) *σ* and (d) *I*.

Fig. 9(a) gives the real and imaginary part of the dielectric constants, *ε*_1_(*ω*) and *ε*_2_(*ω*), respectively. The value of the static dielectric constant *ε*_0_ is 13.4 for silicene. The highest value of *ε*_1_(*ω*) is 5.6 at 3.3 eV. The first peak of *ε*_2_(*ω*) lies at 0.3 eV while its highest peak value is 8.1 at 3.6 eV. The index of refraction *n* is the square root of the dielectric constant. The real part of the index of refraction describes how a wave slows and bends when entering the material, while the imaginary part of the index of refraction, extinction coefficient *k*, describes how a wave gets weaker as it travels. The extinction coefficient calculates the amount of light lost as a result of scattering and absorption for each unit volume. For Si, the measured static refractive index *n*(0) is 3.7 whereas the peak value of *k* is 1.89 and lies in the UV region as shown in Fig. 9(b). High refractive index materials are logically predominant in optoelectronics.

When a specific frequency photon is falling on the target material, the optical conductivity is proportional to the amount of electrons that are released. Fig. 9(c) depicts the highest optical conductivity (*σ*) of 3867 (Ω cm)^−1^ at 3.6 eV for silicene and the first peak value of 1155 (Ω cm)^−1^ at 1.6 eV. The absorption coefficient, which specifies how much light of a certain frequency can penetrate a material before absorption, is used to describe the solar energy conversion efficiency. The materials with higher absorption in the visible region are considered good for solar cell fabrication. The absorption coefficient *I*(*ω*) is initiated at 0 eV. The first peak value is 23.7× 10^4^ cm^−1^ at 1.7 eV and the highest value is 71× 10^4^ cm^−1^ at 3.6 eV as shown in Fig. 9(d).

**Fig. 10:**
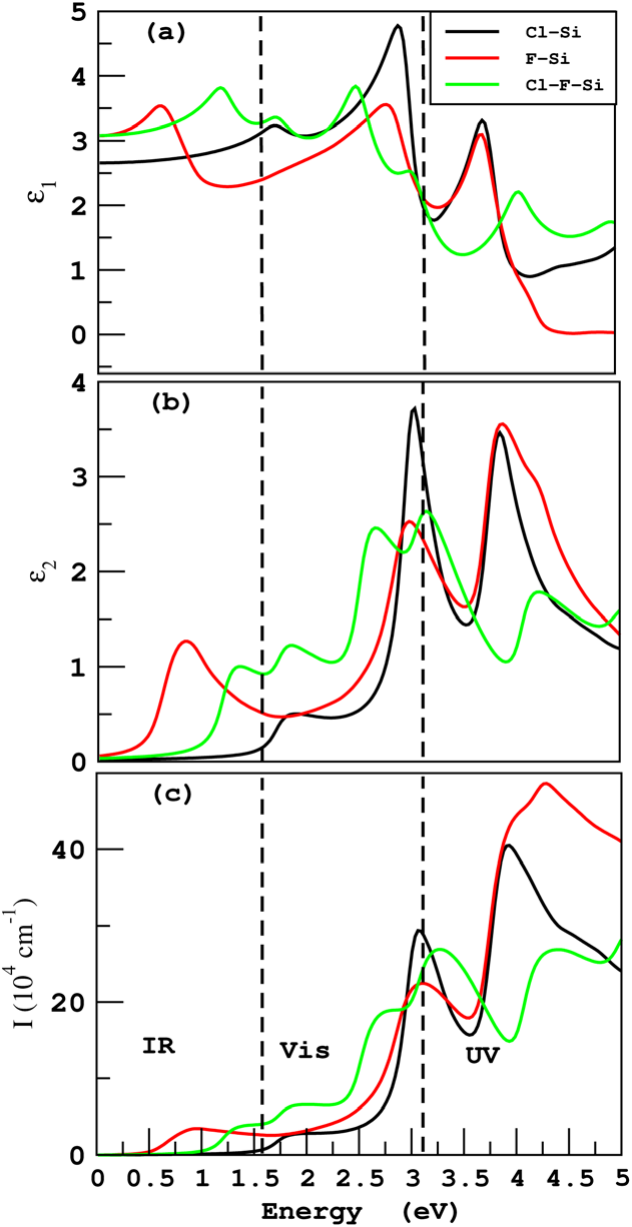
Optical properties of Cl–Si, F–Si and Cl–F–Si, (a) *ε*_1_, (b) *ε*_2_ and (c) *I*.

The frequency-dependent real part *ε*_1_ of a dielectric function, is an indication of the degree to which a material can be polarized. The greater the value of *ε*_1_, the greater the degree of polarization. Fig. 10(a) shows that the frequency-dependent real parts of dielectric function *ε*_1_(*ω*) for Cl–Si, F–Si and Cl–F–Si are 4.7, 3.5 and 3.8, respectively, in the visible region. The static dielectric constant *ε*_1_(0) is the relative permittivity of a material and it is the ratio between permittivity of the material to the permittivity of free space. *ε*_1_(0) for Cl–Si, F–Si and Cl–F–Si are 2.7, 3.0 and 3.08, respectively. The imaginary part *ε*_2_ of a dielectric function describes a material’s ability to absorb energy from a time-varying electric field. The imaginary part is always positive and represents the loss factor or energy absorbed per cubic meter. The first peaks of *ε*_2_(*ω*) for F–Si and Cl–F–Si lie in the infrared region at 1.2 and 2.6, respectively. As far as the highest values of the imaginary parts of the dielectric function in the visible region are concerned, the *ε*_2_(*ω*) for Cl–Si, F–Si and Cl–F–Si are 3.7, 3.5 and 2.6, respectively, as shown in Fig. 10(b). In our case, the electromagnetic waves can travel far in Cl–F–Si due to the low value of *ε*_2_.

How far a specific wavelength of light may go inside a substance before being absorbed is determined by the absorption coefficient *I*. Light will not be effectively absorbed if a substance has a low absorption coefficient, because only the electrons immediately adjacent to the valence band edge are capable of interacting with the photon to generate absorption, the absorption is quite small if the photon has an energy very close to that of the band gap. It is clear from Fig. 10(c), that Cl–Si has the highest value of absorption coefficient *I* in visible region at 30× 10^4^ cm^−1^. While the values of *I* for F–Si and Cl–F–Si are 22× 10^4^ cm^−1^ and 19× 10^4^ cm^−1^, respectively. If we analyze the ultraviolet region, we find that, F–Si has the highest absorption.

If we compare all these results with the optical properties of derivatives of graphene, we find that the derivatives of graphene do not possess absorption in the visible region except chloro–graphene (Cl–C).^15^ While all the derivatives of silicene have remarkable values of absorption in the visible region, which makes them more suitable for optoelectronic devices.

### 3.7 Photo-catalytic properties

**Fig. 11:**
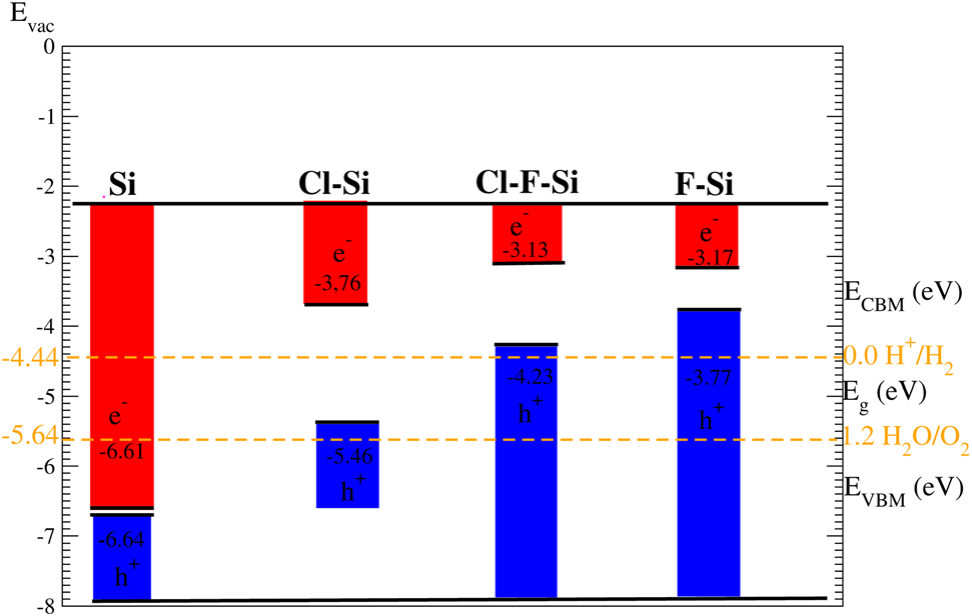
Photocatalytic properties of silicene and its derivatives.

Suitable indirect band gap semiconductors can be used to utilize solar energy to generate hydrogen by dissociating water.^37,38^ Hence photo-catalytic water splitting can be used to clean renewable energy.^39,40^ In the photo-catalytic process, electrons reduce and holes oxidize, water.^41^ The oxidation–reduction potential of 0 (1.23) eV must be small (greater) than the conduction (valence) band for photo-catalytic water splitting; this is investigated for all materials studied here using the Mulliken electronegativity: *E*_VBM_ = −*E*_elec_ + 0.5*E*_g_ and *E*_CBM_ = *E*_VBM_ + *E*_g_^42–44^ as displayed in Fig. 11. On the hydrogen scale the standard oxidation and reduction potentials for photo-catalytic water splitting are −4.44 eV and −5.67 eV, respectively.^45,46^ In order to obtain the band edge positions of the VB and CB with respect to standard oxidation, the Fermi level is set to be −4.44 eV.^47^ The CB and VB are set to 0 eV = −4.44 eV and 1.23 eV = 5.67 eV.^48^ It is clear from Fig. 11 that silicene shows good responses for the reduction of water and all other materials show good response for oxidizing water.

## 4. Conclusion

We have used density functional theory to investigate the structural, electronic and optical properties of silicene and its derivatives. Pristine silicene has a negligible band gap value, thus it cannot be used as a device in photovoltaics, photocatalytic or other semi-conductor-based applications. We have studied the electronic charge density, charge difference density and electrostatic potential which show the ionic bonding characteristic for Si–halides and charge transfer from silicene to Cl/F atoms. We tuned the band gap of silicene by adsorption of the chlorine and fluorine atoms. According to our calculations, the band gap of F–Si is suitable for semiconductor devices. The band gap of Cl–Si and Cl–F–Si is found be very useful for solar cell applications. As far as the TDOS is concerned, in the case of Cl–Si, the Cl atoms provide the major contribution to the VBM, while the Si atoms provide the major contribution to the CBM; while F atoms play a major role in both the VBM and CBM for F–Si. Similarly, F atoms have major contributions to the DOS of Cl–F–Si. Among all the derivatives of silicene, Cl–Si has a maximum absorption of 30× 10^4^ cm^−1^ in the visible region. The materials with higher absorption in the visible region are considered good for solar cell fabrication. Furthermore, we made a comparison of silicene and its derivatives with graphene (C) and its derivatives, chloro–graphene (Cl–C), fluoro–graphene (F–C) and chlorofluoro–graphene (Cl–F–C). The band gap values of all the derivatives of graphene are much higher (ranging from 2.9 eV to 4.9 eV), which makes them unsuitable for semiconducting applications. Calculated binding energies and phonon band structures confirm the stability of Cl–Si, Cl–F–Si, and F–Si. As far as our present work on derivatives of silicene is concerned, the band gap ranging from 0.6 eV to 1.7 eV, is suitable for many potential semiconductor applications.

## Conflicts of interest

The authors declare that they have no known competing financial interests or personal relationships that could have appeared to influence the work reported in this paper.

## Supplementary Material
